# Supplementary material for: Identification of photoperiod-induced specific miRNAs in the adrenal glands of Sunite sheep (Ovis aries)
Source: Front Vet Sci. 2022 Jul 22;9:888207. doi: 10.3389/fvets.2022.888207 (PMC9354845; doi:10.3389/fvets.2022.888207)
Supplement: Supplementary file 1 [file Data_Sheet_1.ZIP › Supplementary file/miRDeep2 file.zip!/miRDeep2 file/Novel_Structure/LP42A2/Novel_33.pdf]

Provisional ID	:	NC_019466.2_17921
Score total	:	2.2
Score for star read(s)	:	-1.3
Score for read counts	:	0
Score for mfe	:	1.9
Score for randfold	:	1.6
Score for cons. seed	:	
Total read count	:	279
Mature read count	:	279
Loop read count	:	0
Star read count	:	0

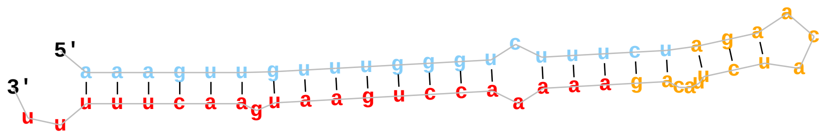

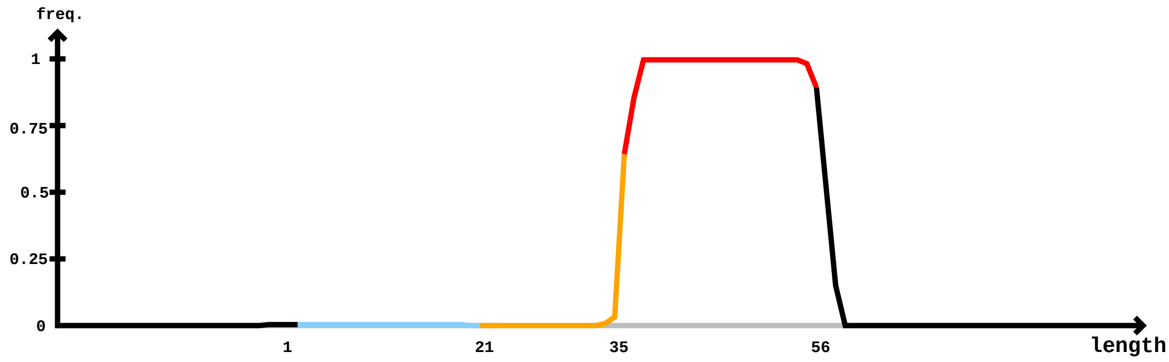

Star Mature

```
5'- acaguaaaauauuggguuggccaaaaaguuguuugggucuuucuagaacaucuuacagaaaaaccugaaugaacuuuuuagccaacccaauaugugaagcaaccgcuuaccu   -3'    exp
    .......(((((((((((((.(((((((((((((((((.((((((((...)))...))))).)))))))).)))))))).)))))))))))))...((((....))))....    reads      mm      sample
    .....................caaaaaguuguuugggucuuu......................................................................    1          0       seq
    ........................................................agaaaaaccugaaugaacuuu...................................    1          0       seq
    ........................................................aAaaaaaccugaaugaacuuuu..................................    1          1       seq
    .........................................................gaaaaaccugaaCgaacuuu...................................    1          1       seq
    .........................................................gaaaaaccugaaugaacuuuA..................................    1          1       seq
    .........................................................gGaaaaccugaaugaacuuuu..................................    1          1       seq
    .........................................................gaaaaaccugaaugaacuuuu..................................    2          0       seq
    .........................................................gGaaaaccugaaugaacuuuuu.................................    2          1       seq
    ..........................................................aaaaaccugaaGgaacuuu...................................    1          1       seq
    ..........................................................aaaaaccCgaaugaacuuuu..................................    2          1       seq
    ..........................................................aaaaaccugaaugaacuuuu..................................    5          0       seq
    ..........................................................aaaaaccugaaCgaacuuuu..................................    5          1       seq
    ..........................................................aaaaaccugaaGgaacuuuu..................................    7          1       seq
    ..........................................................aaaaaccugaaugaacuCuuu.................................    1          1       seq
    ..........................................................aaaaaUcugaaugaacuuuuu.................................    111        1       seq
    ..........................................................aaaaaccugaaGgaacuuuuu.................................    9          1       seq
    ..........................................................aaaaaccugaaugaacuuuuu.................................    19         0       seq
    ..........................................................aaaaaccugaaugaacCuuuua................................    2          1       seq
    ..........................................................aaaaaccugaaugaacuuuuuU................................    4          1       seq
    ..........................................................aaaaaccugaaugaacuuuuua................................    5          0       seq
    ...........................................................aaaaccugaaugaacuuu...................................    1          0       seq
    ...........................................................aaaaccugaaugaacuuuu..................................    1          0       seq
    ...........................................................aaaaccAgaaugaacuuuuu.................................    2          1       seq
    ...........................................................aaaaccugaaugaacuuuuu.................................    6          0       seq
    ...........................................................Gaaaccugaaugaacuuuuu.................................    1          1       seq
    ...........................................................aaaaccCgaaugaacuuuuu.................................    1          1       seq
    ...........................................................Caaaccugaaugaacuuuuu.................................    3          1       seq
    ...........................................................aaaaccugaaugaacuuAuua................................    2          1       seq
    ...........................................................aaaaccugaaugaacuuuuuGg...............................    42         1       seq
    ............................................................aaaccCgaaugaacuuuuu.................................    28         1       seq
    ............................................................aaaccugaaugaacuuuuu.................................    2          0       seq
    ............................................................aaaccCgaaugaacuuuuua................................    10         1       seq
```
